# Chinese Child Venders

**Published:** 1884-12

**Authors:** 


### CHINESE CHILD VENDERS.

A HONG KONG letter says :—In Nankin and Kai-fun children from six to twelve years of age are sold by tens of thousands. Not hired out or transferred—but sold for a small sum in cash, in consideration of which the progenitor, by a tacit understanding, renounces all parental rights, even the right of inquiring into the fate of his offspring. The purchasing trader may be the middle man of a well-to-do childless couple, or the agent of a wholesale tea planter, or coolie breeder, raising and training slaves for a foreign market. For the equivalent of $15 any commission peddler will undertake to "adopt" the same number of young Mongols in the name of any employer, and at very short notice. The authorities might object to a formal and public purchase, but the meaning of the adopting transaction is understood and connived at. It is a lesser evil, and few parents ask any questions. Rather than see their children starve they will resign them to any fate—with one exception. The orthodox Buddhists seem to have evinced occasional scruples in delivering up their yougsters to the proselytizing missionaries, whom they suspect of all sorts of wicked practices. But even such scruples can be readily outweighed by a few extra dollars.

Many Turkish ladies have of late years adopted Western fashions of dress, and have dispensed altogether with the veil, which, according to the twenty-fouth chapter of the Koran, ought to be worn by every Mussulman woman. The Sultan, who is much scandalized by these departures from the ancient usage, has now declared that all his female subjects, under pain of a heavy fine, shall discard their accursed European mantles, their crinolettes, and their Parisian boots, and that henceforth every Mahomedan woman shall shroud her head in a veil of modest capacity, and clothe herself in all ways after the decent fashion of her ancestors.

**Color-Blindness.**—An amusing story is told of the great English chemist, Dalton, who was red-blind and also a Quaker. When about to be presented at court, he learned that he must wear a scarlet coat as part of his court-dress. This he refused to do from conscientious scruples. But his friends were determined that he should be presented, and, knowing his infirmity, dressed him in a coat of the regulation shade, gravely informing him that it was of a dark hue ; and, as he was unable to tell the difference, he wore the objectionable color without being aware that he was violating his religious principles.

